# FastRFS: fast and accurate Robinson-Foulds Supertrees using constrained exact optimization

**DOI:** 10.1093/bioinformatics/btw600

**Published:** 2016-09-23

**Authors:** Pranjal Vachaspati, Tandy Warnow

**Affiliations:** 1 Department of Computer Science, University of Illinois, Urbana-Champaign, Urbana, IL, USA; 2 National Center for Supercomputing Applications, University of Illinois, Urbana-Champaign, Urbana, IL, USA; 3 Department of Bioengineering, University of Illinois, Urbana-Champaign, Urbana, IL, USA

## Abstract

**Motivation:**

The estimation of phylogenetic trees is a major part of many biological dataset analyses, but maximum likelihood approaches are NP-hard and Bayesian MCMC methods do not scale well to even moderate-sized datasets. Supertree methods, which are used to construct trees from trees computed on subsets, are critically important tools for enabling the statistical estimation of phylogenies for large and potentially heterogeneous datasets. Supertree estimation is itself NP-hard, and no current supertree method has sufficient accuracy and scalability to provide good accuracy on the large datasets that supertree methods were designed for, containing thousands of species and many subset trees.

**Results:**

We present FastRFS, a new method based on a dynamic programming method we have developed to find an exact solution to the Robinson-Foulds Supertree problem within a constrained search space. FastRFS has excellent accuracy in terms of criterion scores and topological accuracy of the resultant trees, substantially improving on competing methods on a large collection of biological and simulated data. In addition, FastRFS is extremely fast, finishing in minutes on even very large datasets, and in under an hour on a biological dataset with 2228 species.

**Availability and Implementation:**

FastRFS is available on github at https://github.com/pranjalv123/FastRFS

**Supplementary information:**

Supplementary data are available at *Bioinformatics* online.

## 1 Introduction

Supertree estimation is the problem of computing a tree on a set *S* of taxa from a set of estimated trees (called ‘source trees’) on subsets of *S*. Traditionally, the purpose of supertree estimation was to combine published species trees estimated by different research groups around the world, using different datasets and different methods. Supertree methods have been used to construct many species trees, and the development of supertree methods is an area of very active research (see Bininda-Emonds, 2004 for some of the early literature, and Akanni *et al.,* 2014; Martins *et al.,* 2016; Nguyen *et al.,* 2012; Swenson *et al.,* 2012 for some more recent methods).

More recently, supertree estimation has been used within divide-and-conquer frameworks, in which a large and potentially heterogeneous dataset is divided into overlapping smaller subsets, trees are estimated on each subset, and then combined into a tree on the full dataset using a supertree method. These divide-and-conquer methods thus enable the application of statistical phylogeny estimation methods to scale to larger datasets (Bayzid *et al.*, 2014; Huson *et al.*, 1999; Nelesen *et al.*, 2012; Warnow *et al.*, 2001). Each of these methods has been able to improve the accuracy and/or speed of its base method. Thus, supertree computation provides an essential tool for both moderate- and large-scale phylogeny estimation, and is relevant to both gene tree estimation and species tree estimation.

One of the popular approaches to supertree estimation is the NP-hard Robinson-Foulds Supertree problem (Bansal *et al.*, 2010), which seeks a binary tree that has the minimum total Robinson-Foulds (Robinson and Foulds, 1981) distance to the input source trees. The best known local search heuristic for the Robinson-Foulds Supertree is MulRF (Chaudhary *et al.*, 2014), but PluMiST (Kupczok, 2011) is a new method that shows promise; to our knowledge, there are no other methods that are competitive with these two.

One of the exciting properties of the Robinson-Foulds Supertree problem is that it is closely related to the Maximum Likelihood Supertree problem, which seeks a supertree that is the most likely to have produced the observed source trees under a simple exponential model of phylogenetic error (Steel and Rodrigo, 2008). Although the two problems are not identical (as established in Bryant and Steel, 2009), it seems likely that good solutions to the Robinson-Foulds Supertree problem will be good solutions to the Maximum Likelihood Supertree problem. However, the only technique for the Maximum Likelihood Supertree problem that we are aware of, L.U.-st (Akanni *et al.*, 2014), is very computationally intensive, making it infeasible for use on biological datasets (Akanni *et al.*, 2015b).

In this paper, we report on a new method, FastRFS (Fast Robinson-Foulds Supertrees) for finding optimal Robinson-Foulds Supertrees in a constrained search space. Unlike the previous methods for Robinson-Foulds Supertrees, which depended on heuristic searches through tree space, the method we have designed uses dynamic programming (DP) to find an exact solution to the Robinson-Foulds Supertree problem within a constrained search space.

This algorithmic strategy of using dynamic programming to find a species tree optimizing some criterion within a constrained search space was first used in Hallett and Lagergren (2000); since that time, the approach has been used in other phylogenetic estimation methods (Bayzid *et al.*, 2013; Bryant and Steel, 2001; Mirarab and Warnow, 2015; Mirarab *et al.*, 2014; Than and Nakhleh, 2009; Yu *et al.*, 2011). Most of these methods constrain the search space for their optimization problem by computing a set *X* of allowed bipartitions (i.e. splits of the leafset into two parts, each defined by deleting edges in the species tree that will be constructed) from the input, and require that the output tree draw its bipartitions from *X*. These methods run in time that is polynomial in the number of species, source trees, and |X|. Many of these methods specify *X* to be the set of bipartitions in the input source trees, but expanding the set can improve accuracy (Mirarab and Warnow, 2015).

The supertree method we present, FastRFS, is a combination of the polynomial time dynamic programming algorithm for the constrained Robinson-Foulds Supertree problem we have developed and the technique we use to define the set *X* from the input source trees. The basic FastRFS method uses ASTRAL-2 to define the set *X* of allowed bipartitions from the input set of source trees. We also explore an enhanced version where we add additional bipartitions (beyond those computed by ASTRAL-2) to the set X defined by ASTRAL-2. We define the additional bipartitions by computing fast supertrees on the input set, and then add their bipartitions to *X*; this approach ensures that we find RFS criterion scores that are at least as good as the trees we use to define the set *X* of allowed bipartitions, and also at least as good as the trees obtained by the basic FastRFS method. By only adding bipartitions from supertrees that we can compute quickly, the enhanced FastRFS method is able to complete quickly, and provides improved criterion scores.

We evaluate these two versions of FastRFS in comparison to leading methods for supertree estimation on a collection of biological and simulated datasets with 100–2228 species that were used in prior publications to evaluate supertree methods (Nguyen *et al.*, 2012; Swenson *et al.*, 2010, 2012). We compare FastRFS to PluMiST, the current best performing method (in terms of criterion scores) for the Robinson-Foulds Supertree problem, and also to MulRF, the most well-known software for this optimization problem. We also compare FastRFS to Matrix Representation with Likelihood (MRL) (Nguyen *et al.*, 2012), ASTRID (Vachaspati and Warnow, 2015) and ASTRAL-2 (Mirarab and Warnow, 2015). MRL is the maximum likelihood counterpart to the well-known Matrix Representation with Parsimony (MRP) method, and has produced topologically more accurate supertrees than leading MRP heuristics (Nguyen *et al.*, 2012). ASTRID and ASTRAL-2 are methods for species tree estimation that take gene tree heterogeneity arising from incomplete lineage sorting into account, and have had good accuracy on large phylogenomic datasets. We evaluate these methods with respect to RFS criterion scores (which can be evaluated on both simulated and biological datasets), topological accuracy in estimating the true supertree (which can only be evaluated on simulated datasets) and wall clock running time.

## 2 Materials and methods

Every model tree and estimated supertree in this study is a fully resolved tree, and no two leaves have the same label; the source trees are unrooted trees with leaves drawn from (possibly proper) subsets of the full set of taxa, and may contain polytomies (nodes of degree greater than three). We let T|Q denote the tree obtained by restricting the tree *T* to the subset *Q* of its leafset, and then suppressing nodes of degree two. We let L(T) denote the leafset of a tree *T*. The deletion of an edge *e* from a tree *T* induces a bipartition of L(T) into two sets *A* and *B*, denoted by [A,B]. Every unrooted tree *T* is defined by its set *Bip*(*T*) of bipartitions. The Robinson-Foulds (RF) distance between trees *T* and T′ that are on the same leafset is the number of bipartitions that are in one tree but not the other (i.e. RF(T,T′)=|Bip(T)△Bip(T′)|). Note that when *T* and T′ have the same leafset, then RF(T,T′)=0 if and only if T=T′.

We extend the definition of RF distance to trees *t* and *T* with nested leafsets (i.e. L(t)⊆L(T)) to be the RF distance between T|L(t) and *t*, and denote this distance by *RF*(*T*, *t*). Given a set T of trees and tree *T* satisfying L(t)⊆L(T) for all t∈T, we define $RF(T,T)=\sum_{t\in T}RF(T,t).$ A binary tree *T* with leafset $S=\cup_{t\in T}L(t)$ that minimizes RF(T,T) is the Robinson-Foulds Supertree for T, and is denoted *T_RFS_*.

Finding a Robinson-Foulds Supertree is NP-hard; however, the Constrained Robinson-Foulds Supertree Problem constrains the search space using a set *X* of allowed bipartitions, and can be solved in polynomial time, as we will show.

*Constrained Robinson-Foulds Supertree Problem:*

- Input: Set T of trees and set *X* of bipartitions of the taxon set *S*, where $S=\underset{t\in T}{\cup }L(t)$.
- Output: Unrooted binary tree $T_{RFS(c)}$ that minimizes RF(T,T), subject to the constraint that every bipartition in $T_{RFS(c)}$ is drawn from *X*.

### 2.1 The dynamic programming algorithm to solve constrained Robinson-Foulds supertrees

While the Robinson-Foulds Supertree problem is stated in terms of minimizing the total Robinson-Foulds distance to the source trees, we will rephrase it as *maximizing the bipartition support* from the source trees. This formulation will make it easy for us to present and explain the dynamic programming approach we have developed.

Let *t* be a source tree with leafset S′ and let *T* be a tree with leafset *Y*, so that S′⊆Y⊆S. Let [A′,B′] be a bipartition in *t*. We will say that [A′,B′] supports *T* if there is a bipartition [A,B] in *T* such that A′=S′∩A and B′=S′∩B. We will also say that the bipartition support of *t* for *T* is the number of bipartitions in *t* that support *T*, and that the bipartition support from T for *T* is the bipartition support for *T* from all the trees in T.

Observation 1 *For any set*T*of source trees, a binary tree T with leafset*$S=\cup_{t\in T}L(t)$*that has the maximum bipartition support from*T*is an optimal solution to the Robinson-Foulds Supertree problem*.

Recall that the input includes a set *X* of allowed bipartitions. A clade in a rooted tree is a set of leaves that constitute all the leaves below some selected node in the rooted tree. We define a set C of allowed clades, by setting C={A:∃[A,B]∈X} (i.e. C contains every half of every bipartition in *X*).

Let *t* be an unrooted tree with leafset S′, let *T* be a rooted binary tree with leafset *Y* where S′⊆Y, and let [A′,B′] be a bipartition in *t*. We will say that [A′,B′] supports *T* if T|S′ contains A′ or B′ (or both) as clades. We define the bipartition support of source tree t∈T for the rooted tree *T* to be the number of bipartitions in *t* that support *T*, and the bipartition support of T for *T* to be the total of the bipartition support from all the source trees in T for *T*. Furthermore, given node *v* in *T*, we let *T_v_* denote the subtree of *T* rooted at *v*; note that every node in *T_v_* is also a node in *T*.

Observation 2 *For all sets*T*of source trees and all rooted trees T with leafset*$S=\cup_{t\in T}L(t)$*, the bipartition support of*T*for T is the same as the bipartition support of*T*for the unrooted version of T*.

By Observation 2, we can solve the Constrained Robinson-Foulds Supertree problem by finding a rooted tree with leafset *S* that has the maximum bipartition support, and then unrooting this tree.

For the rest of this discussion, *T* will denote a rooted binary tree with leafset Y⊆S, with all its clades drawn from C. We will show that we can write the bipartition support for *T* from a source tree *t* as the sum of the bipartition support for the clades in *T*, which will allow us to construct a dynamic programming algorithm.

Consider an internal node *v* in *T*, and let *v*_1_ and *v*_2_ be its two children. Let the clade below *v* be *A*, the clade below *v*_1_ be *A*_1_, and the clade below *v*_2_ be *A*_2_. Deleting *v* from *T* splits *Y* into three parts: *A*_1_, *A*_2_ and $A_{3}=Y\setminus A$. We will describe this by saying *v* defines the ordered tripartition $\left(A_{1},A_{2},A_{3}\right)$, with the understanding that $\left(A_{1},A_{2},A_{3}\right)$ and $\left(A_{2},A_{1},A_{3}\right)$ are equivalent, and both correspond to node *v*. Note that if Y≠S, then the tripartition defined by *v* will not cover all the elements of *S*. Also, we will require that *A*_1_ and *A*_2_ be allowed clades (i.e. in C), but we make no such constraint on *A*_3_.

Suppose that source tree *t* with leafset S′ has a bipartition [U′,V′] that supports *T*; thus, T|S′ must have U′ or V′ (or both) as clades. We wish to associate this bipartition to exactly one node in *T*, so that we can compute the bipartition support without having to correct for over-counting, and so that the dynamic programming algorithm is simple.

Case 1: T|S′ contains only one of these two clades. Suppose T|S′ contains U′ but not V′ as a clade. If T|S′ does not contain any leaves from V′, we do *not* assign [U′,V′] to any node in *T*. If T′|S′ does contain at least one leaf from V′, we follow the path from the MRCA of U′ towards the root until we reach the first node *w* that has at least one element of V′ in the subtree below it, and we assign [U′,V′] to *w*.
Case 2: T|S′ contains both U′ and V′ as clades. We assign [U′,V′] to the MRCA of U′∪V′.

The following lemma follows directly from the description of the assignment process:

Lemma 1 *For any bipartition*π=[U′,V′]*and any tree T, π is assigned to node w in T if and only if w defines a tripartition*$\left(A_{1},A_{2},A_{3}\right)$*where*$U\prime \subseteq A_{1},V\prime \cap A_{1}=\emptyset ,$*and*$V\prime \cap A_{2}\neq \emptyset$*. If π supports T, then there is a unique node in T satisfying this constraint. However, if no such node exists, π does not support T, and so is not assigned to any node in T.*

Lemma 2 *Let T be a rooted tree on set Y, and let v be a node in T other than the root. Let*[U′,V′]*be a bipartition in a source tree t that supports both T and T_v_, and suppose that*[U′,V′]*is assigned to node w in T and node*w′*in T_v_. Then*w=w′.

Proof By Lemma 1, [U′,V′] is assigned to the unique node w′ in *T_v_* that defines a tripartition $\left(A_{1},A_{2},A_{3}\right)$ where $U\prime \subseteq A_{1},V\prime \cap A_{1}=\emptyset ,$ and $V\prime \cap A_{2}\neq \emptyset$. Since *T_v_* is a rooted subtree of *T*, the node w′ exists in *T*, and defines the tripartition $\left(A_{1},A_{2},A_{3}^{\prime }\right)$ that differs from the tripartition above only in the third coordinate. By Lemma 1, it follows that w=w′. □

Note that the assignment of bipartitions to nodes in trees depends only on the first two components of the tripartition for the node. We make the following definition:

Definition 1 *Let A_1_, A_2_ be a disjoint pair of allowed clades. We define*$support(A_{1},A_{2})$*to be the number of bipartitions in the source trees that map to a tripartition*$\left(A_{1},A_{2},Z\right)$*for some Z.*

Theorem 1 *The bipartition support from*T*for a rooted binary tree T is*

$$\sum_{\left(A_{1},A_{2},A_{3})\in Trip(T\right)}support(A_{1},A_{2}),$$

*where Trip(T) denotes the set of tripartitions defined by the nodes of T*.

Proof The prior discussion establishes that for a given source tree t∈T and bipartition $\pi_{e}\in Bip(T)$ that supports *T*, there is exactly one tripartition in *Trip*(*T*) that π_*e*_ is mapped to. Furthermore, if π_*e*_ does not support *T*, then it is not mapped to any tripartition in *Trip*(*T*). The theorem follows. □

Theorem 2 *Let*T*be a set of source trees with S the set of taxa that appear as a leaf in at least one tree in*T*, and let*C*be the set of allowed clades. Set*BPS({s})=0*for all*s∈S*, and let BPS(A) for*A∈C*with*|A|≥2*be the maximum bipartition support over all rooted binary trees T on clade A where T draws its clades from*C*. Then, for*A∈C,|A|≥2,

(1) $$BPS(A)=\max\{BPS(A_{1})+BPS(A_{2})+support(A_{1},A_{2}):A=A_{1}\cup A_{2},A_{1}\cap A_{2}=\emptyset ,A_{i}\in C\}$$

Proof Let A∈C be arbitrary, with |A|≥2. Let $BPS^{*}(A)$ denote the maximum achievable bipartition support of any rooted tree on *A* that draws its clades from C, and let *BPS*(*A*) be the value as computed by Equation 1. We will prove by induction on the size of *A* that $BPS^{*}(A)=BPS(A)$.

The base case is A={a,a′}. There is only one rooted tree on *A*, and it has bipartition support support({a},{a′}), which is equal to *BPS*(*A*). Hence $BPS^{*}(A)=BPS(A)$ for |A|≤2. Now let |A|>2 be arbitrary, and let *T* be a binary rooted tree with leafset *A* having the largest bipartition support from the trees in T, and drawing its clades from C. The inductive hypothesis is that $BPS(A\prime )=BPS^{*}(A\prime )$ for all proper subsets A′ of *A* where A′∈C.

Let *v*_1_ and *v*_2_ be the two children of the root of *T*, *A*_1_ and *A*_2_ be the leafsets of the subtrees of *T* rooted at *v*_1_ and *v*_2_, and *T*_1_ and *T*_2_ be the subtrees of *T* rooted at *v*_1_ and *v*_2_, respectively. By the inductive hypothesis, $BPS(A_{1})=BPS^{*}(A_{1})$ and $BPS(A_{2})=BPS^{*}(A_{2})$. Because *T* optimizes the bipartition support of all rooted binary trees on *A* given the constraint set, *T*_1_ and *T*_2_ have the highest bipartition support of all rooted binary trees on *A*_1_ and *A*_2_, respectively, given the constraint set. By Theorem 1, the bipartition support of *T_i_* is the sum of *support*(*X*, *Y*) for all tripartitions defined by the nodes of *T_i_*, for *i* = 1, 2, and the bipartition support of *T* is the sum of *support*(*X*, *Y*) for all tripartitions defined by the nodes of *T*. Hence, the bipartition support of *T* is $BPS(A_{1})+BPS(A_{2})+support(A_{1},A_{2})$. Thus, $BPS^{*}(A)=BPS(A_{1})+BPS(A_{2})+support(A_{1},A_{2})$, and so $BPS^{*}(A)\leq BPS(A)$.

To complete the proof, we need only show that $BPS(A)\leq BPS^{*}(A)$. So suppose $BPS(A)>BPS^{*}(A)$. Then there is a bipartition $\left[A\prime_{1},A\prime_{2}\right]$ of *A* such that $BPS(A\prime_{1})+BPS(A\prime_{2})+support(A\prime_{1},A\prime_{2})>BPS(A_{1})+BPS(A_{2})+support(A_{1},A_{2})$. Let $T\prime_{1}$ and $T\prime_{2}$ be the rooted trees on $A\prime_{1}$ and $A\prime_{2}$ having quartet support $BPS^{*}(A\prime_{1})$ and $BPS^{*}(A\prime_{2})$, respectively, with clades drawn from C, and let T′ be the binary rooted tree on *A* with subtrees $T\prime_{1}$ and $T\prime_{2}$. Then T′ draws its clades from C and has bipartition support that is strictly greater than that of *T*. This contradicts the assumption that *T* had the largest bipartition support among all rooted binary trees drawing its clades from C. Hence, $BPS(A)\leq BPS^{*}(A)$. We have shown that $BPS(A)\leq BPS^{*}(A)$ and $BPS^{*}(A)\leq BPS(A)$, and so $BPS(A)=BPS^{*}(A)$. Since *A* was arbitrary, the theorem follows. □

### 2.2 The dynamic programming algorithm

The input is a pair (T,X) where T is a set of source trees and *X* is a set of allowed bipartitions.

- Preprocessing: Compute the set C of allowed clades, and order them by cardinality from smallest to largest. Compute the set *S* of taxa. Set BPS({s})=0 for all s∈S. Compute $support(A_{1},A_{2})$ for every pair of disjoint allowed clades *A*_1_, *A*_2_.
- For each A∈C with |A|≥2, in order of size (from smallest to largest), set
  $$BPS(A)=\max\{BPS(A_{1})+BPS(A_{2})+support(A_{1},A_{2})\},$$

where *A*_1_ and *A*_2_ are disjoint allowed clades and $A=A_{1}\cup A_{2}$.

- Return *BPS*(*S*).
- Compute a rooted binary tree achieving this score using backtracking, and then unroot it to produce a Robinson-Foulds Supertree.

Theorem 3 The dynamic programming algorithm finds an optimal solution to the constrained Robinson-Foulds Supertree problem, and does so in $O(|X{|}^{2}nk)$ time, where there are n taxa and k source trees.

Proof Let (T,X) (where T is the set of source trees and *X* is a set of bipartitions on the species set *S*) be an input to the constrained Robinson-Foulds supertree problem, and let C be the set of halves of these bipartitions. By Theorem 2, the dynamic programming algorithm correctly computes the best achievable bipartition support for any rooted supertree drawing its clades from set C. Backtracking produces a rooted *T* achieving that optimal score, and unrooting *T* produces T′, which has the same optimal score. By construction, *T* draws its clades from C, and so T′ draws its bipartitions from *X*. Hence, the output from the algorithm, T′, is a supertree that draws its bipartitions from *X* and that achieves the best possible bipartition support score of all supertrees drawing their bipartitions from *X*; this establishes correctness.

For the running time analysis, we begin with the preprocessing step. Note that |C|=2|X| and that there are *O*(*nk*) bipartitions in the source trees. For each of the O(|X|) allowed clades *A* and each half *Y*_1_ of the *O*(*nk*) source tree bipartitions, we determine if $Y_{1}\subseteq A$; this takes *O*(*n*) time per comparison, for a total cost of $O(|X|n^{2}k)$ time. Once this is done, we can compute $support(A_{1},A_{2})$ for every pair *A*_1_, *A*_2_ of disjoint allowed clades, using $O(|X{|}^{2}nk)$ additional time. Since |X|≥n−3, $|X|n^{2}k\leq |X{|}^{2}nk$; hence, the preprocessing is done in $O(|X{|}^{2}nk)$ time. The second phase, where we compute *BPS*(*A*) for the allowed clades *A*, is easily seen to take O(|X|) time per clade, provided that the preprocessing is done first, and the calculations are done in the proper order. Hence, the total time is $O(|X{|}^{2}nk)$ time. □

### 2.3 The basic FastRFS method

The input to the FastRFS method is a set T of unrooted source trees, but they do not need to be binary trees (i.e. polytomies are allowed). In the basic FastRFS method, we use ASTRAL-2 to compute the set *X* of allowed bipartitions. The technique in ASTRAL-2 for computing the set *X* of allowed bipartitions produces a set that contains at least one compatible subset of *n* – 3 bipartitions, where n=|S|; as a result, FastRFS is guaranteed to return a fully resolved tree on every input. See Mir arabbaygi (Mirarab) (2015) for details on how ASTRAL-2 computes the set *X*.

### 2.4 FastRFS-enhanced and ASTRAL-enhanced

The enhanced version of FastRFS, which we write as FastRFS-enhanced, operates by computing a set *Z* of supertrees that can be computed quickly on T, and then adds the bipartitions from trees in *Z* to the set *X* that is computed by ASTRAL-2. This approach ensures that the RFS criterion score found by FastRFS-enhanced will be *at least as good* as any tree in *Z*.

In our study, we used one or both of ASTRID and MRL for our set *Z*. ASTRID computes a matrix of average pairwise ‘internode distances’ (the number of edges in the path between two species in a tree), and then computes a tree on the distance matrix. When the distance matrix has no missing data, ASTRID uses FastME (Desper and Gascuel, 2002), a fast and accurate method to compute the supertree; however, when the distance matrix has missing entries, it uses BioNJ* (Criscuolo and Gascuel, 2008), a method that is slower and not quite as accurate (see Vachaspati and Warnow, 2015 for a comparison of ASTRID using BioNJ* and ASTRID using FastME). In our experiments, we include the MRL tree in *Z*, and we also include the ASTRID tree for those inputs where the internode distance matrix has no missing entries. We similarly define ASTRAL-enhanced using the same set of extra trees as for FastRFS-enhanced.

### 2.5 Datasets

We used a collection of published simulated and biological datasets that have been used in other studies (Swenson *et al.*, 2010) to evaluate supertree methods, all of which are available online at http://www.cs.utexas.edu/users/phylo/datasets/supertrees.html.

The simulated data, referred to as ‘SMIDgen’ in Swenson *et al.* (2010), are generated using a taxon sampling strategy that mimics biological practice. These datasets have 100, 500, or 1000 taxa, with up to 25 source trees per replicate. Each supertree input has several ‘clade-based’ source trees and a ‘scaffold tree’, which are estimated using maximum likelihood heuristics on a concatenation of gene sequence alignments. Some genes are ‘universal’ and so are present in every species; others evolve within the species tree under a birth-death model in which birth happens once but death (i.e. gene disappearance) can happen several times; therefore, unless the gene is born at the root of the species tree, it will be present only within a clade within the tree. Sequences then evolve down the gene trees under the GTRGAMMA model of site evolution. The scaffold tree is based only on the universal genes, and has a random subset of the species set; the clade-based trees are obtained by selecting a clade in the tree and then a set of genes that covers that clade well. As shown in Swenson *et al.* (2010), the density of the scaffold tree (i.e. the percentage of the full set of taxa that are in the scaffold dataset) has a large impact on the topological accuracy of the resultant estimated supertree. These simulated data enable us to evaluate topological accuracy as well as criterion score.

We include the biological datasets that were also studied in Swenson *et al.* (2010): CPL (comprehensive papilinoid legumes) (McMahon and Sanderson, 2006), Marsupials (Cardillo *et al.*, 2004), Placental Mammals (Beck *et al.*, 2006), Seabirds (Kennedy *et al.*, 2002) and THPL (temperate herbaceous papilionmoid legumes) (Wojciechowski *et al.*, 2000). These range in size from 116 species (Placental Mammals) to 2228 (CPL), and with as few as 7 source trees (Seabirds) to as many as 726 (Placental Mammals).

### 2.6 Methods

In addition to the two FastRFS variants (basic and enhanced), we computed supertrees using MRL, ASTRID, ASTRAL-2, MulRF and PluMiST. For MRL, we compute the MRP matrix using ‘mrpmatrix’ available at github.com/smirarab/mrpmatrix, and we use RAxML (Stamatakis, 2014) version 8.2.4 under the BINGAMMA model with seed 12345 on the MRP matrix. We ran MulRF version 1.2 (Chaudhary *et al.*, 2014) and PluMiST version 1.1 (Kupczok, 2011). We ran PluMiST in default mode, and we ran MulRF ten times, and report results for the tree with the best criterion score. We ran ASTRAL-2 version 4.7.12 (Mirarab and Warnow, 2015) (henceforth referred to as ASTRAL) and ASTRID version 1.1 (Vachaspati and Warnow, 2015), both in default mode. Each of these methods produces fully resolved unrooted trees.

ASTRAL produces a supertree that minimizes the total quartet distance to the input source trees (equivalently, it produces a supertree that maximizes the total quartet tree support) subject to a constrained set *X* of bipartitions that it computes from the input source trees.

We tested an enhanced version of ASTRAL (analogous to FastRFS-enhanced), in which we added the bipartitions from MRL and ASTRID to the set *X*; this enables a direct comparison of FastRFS-enhanced and ASTRAL-enhanced. Although FastRFS-enhanced is guaranteed to find RFS criterion scores that are at least as good as ASTRAL-enhanced, the comparison with respect to tree topology accuracy makes it possible to evaluate the two optimization criteria (minimize quartet distance or minimize Robinson-Foulds distance) and their impact on topological accuracy. Finally, we tested the impact of adding the bipartitions from just one tree (MRL or ASTRID) to the set *X* on FastRFS, to determine the relative impact of each additional tree.

### 2.7 Measurements

We can use the simulated data to explore performance with respect to criterion scores as well as tree estimation error. However, since there is no known true supertree for the biological datasets, we use the biological datasets to explore performance only with respect to criterion scores.

For tree estimation error (explored only on the simulated datasets), we report the normalized bipartition distance (also called the Robinson-Foulds error rate) between the estimated and true trees. The Robinson-Foulds (RF) error rate is $\frac{RF(T,T\prime )}{2n-6}$, where RF(T,T′) is the RF distance between the true tree *T* and the estimated tree T′, and *n* is the number of leaves in *T*). Hence, the RF error rate is between 0 and 1, and is equal to 0 if and only if the two trees are identical.

We also report the Robinson-Foulds Supertree criterion score (i.e. the total Robinson-Foulds distance between the estimated supertree and the input source trees) for all datasets; this value is bounded from above by (2n−6)k, where *n* is the total number of species and *k* is the total number of source trees.

Although the criterion scores and tree error metrics both refer to the Robinson-Foulds distance, the criterion score is based on the RF distance to the input source trees, and the tree error metric refers to the RF distance to the model tree, which is unknown. Hence these are two different ways of evaluating methods.

Most of the methods are sequential codes; however, FastRFS is parallelized to run on 8 cores and we run MulRF 10 times in parallel and take the best tree. We report wall clock running times for all codes; except when the differences are large, comparisons between running times are not reliable. Running times for FastRFS-enhanced include the time to compute the MRL tree and the ASTRID distance matrix, and, if the distance matrix has no missing data, the time to run FastME on the distance matrix (i.e. to fully compute the ASTRID tree).

### 2.8 Experiments

We performed experiments to evaluate the different supertree methods with respect to Robinson-Foulds criterion score, topological accuracy of the supertree and running time.

## 3 Results and discussion

### 3.1 Impact of the constraint set on criterion scores

Our initial experiment evaluated the impact on the criterion scores found by FastRFS of adding bipartitions from the MRL tree and/or the ASTRID tree to the constraint set. In general, FastRFS with the MRL tree alone added was nearly as good as FastRFS-enhanced (i.e. with both ASTRID and MRL trees added), and FastRFS with MRL found substantially better criterion scores than FastRFS with just the ASTRID tree added. Nevertheless, since adding the ASTRID tree did help occasionally, and since ASTRID is so quick to run when the distance matrix is complete, we continued using it for FastRFS-enhanced. See supplementary materials for these results.

### 3.2 Criterion scores for the simulated datasets

By design, FastRFS-enhanced will always find criterion scores that are at least as good as those found by ASTRAL-enhanced, FastRFS-basic, ASTRAL and MRL. Hence, the only methods that could possibly find better scores than FastRFS-enhanced are PluMiST, MulRF and ASTRID. We show the Robinson-Foulds Supertree criterion scores in Table 1; note that lower is better. PluMiST failed to complete on three datasets (one replicate in the 100-taxon and two replicates in the 500-taxon datasets, each with 20%-scaffolds); we report results only on the remaining datasets. Both PluMiST and MulRF had very large running times on the 500-taxon datasets; therefore, we did not attempt to run them on the 1000-taxon datasets. All other methods succeeded in completing on all datasets we examined.

**Table 1:** Average Robinson-Foulds Supertree criterion scores on the simulated datasets; lower is better

Method	100	100	100	100	500	500	500	500	1000	1000	1000	1000
Scaffold %	20	50	75	100	20	50	75	100	20	50	75	100
# Replicates	9	10	10	10	8	10	10	10	10	10	10	10
ASTRAL	32	31	38	45	170	190	225	274	365	414	502	591
ASTRAL-enhanced	32	30	38	45	163	182	221	274	337	393	491	591
ASTRID	40	41	50	41	360	914	905	223	1066	2447	2370	470
MRL	30	30	36	42	158	179	202	223	309	362	412	474
MulRF	32	34	38	**40**	282	315	279	229	–	–	–	–
PluMiST	31	29	**34**	**40**	210	245	246	214	–	–	–	–
FastRFS-basic	**29**	29	**34**	**40**	152	173	191	209	325	366	394	434
FastRFS-enhanced	**29**	**28**	**34**	**40**	**148**	**166**	**186**	**206**	**292**	**347**	**384**	**426**

No results shown for the 1000-taxon datasets for MulRF and PluMiST, due to time constraints; otherwise, results are shown for those datasets for which all methods completed. The best result shown for a given model condition is boldfaced.

FastRFS-enhanced found the best (lowest) Robinson-Foulds Supertree (RFS) criterion scores of all methods for all datasets; FastRFS-basic also found these best scores for three of the four 100-taxon model conditions, but otherwise found higher scores. PluMiST found better RFS criterion scores than MulRF in 7 of the 8 model conditions, and matched in 1 condition. ASTRID had the worst performance of all methods, with much larger criterion scores on all the sparse scaffold model conditions. These are the same conditions in which the internode distance matrix has missing entries, suggesting that the reduced accuracy is largely due to the missing data in the distance matrix.

Certain additional trends are worth noting. First, although PluMiST did well on the 100-taxon datasets, it was not so competitive with FastRFS-enhanced or even FastRFS-basic on the 500-taxon datasets, suggesting that the number of taxa may impact the ability of PluMiST to find trees with good criterion scores. ASTRAL-enhanced matched or improved on the RFS criterion scores compared to ASTRAL; this is interesting because it does not follow from the algorithm design (the two methods seek the tree that minimizes the quartet distance, not the RFS criterion). MRL, although never coming in first, often had very good results, coming just behind FastRFS-basic for overall performance.

### 3.3 Criterion scores on biological datasets

**Fig. 1:**
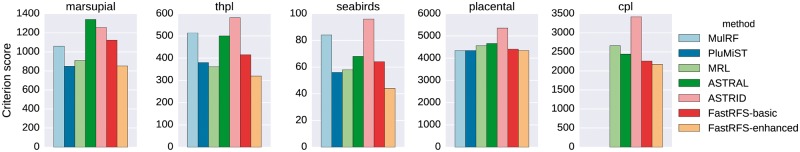
RFS criterion scores on biological data of supertree methods; lower is better. MulRF and PluMiST could not be run on the CPL dataset due to its large size; hence no values are shown for those methods on that dataset. Overall, FastRFS-enhanced produces the best RFS criterion scores on these datasets

We were unable to run PluMiST and MulRF on the CPL dataset, the largest in our collection, due to its size: at 2228 species, the running time needed for these two methods is excessive. Criterion scores on the biological datasets follow very similar patterns as observed on the simulated datasets (Fig. 1). Overall, FastRFS-enhanced had the best criterion scores: the best on four datasets, and close to best on the last dataset (Marsupials). PluMiST tied for best with FastRFS-enhanced on two of the four datasets on which it can run, had the second best score on seabirds, and third best on THPL. Hence, PluMiST is in second place. Interestingly, the dataset on which PluMiST was not able to find one of the top two scores was the second largest dataset, with more than 500 species. Thus, just as we saw on the simulated datasets, the number of species seems to impact the relative performance of PluMiST in comparison to other methods.

The next two best methods are MRL and FastRFS-basic, which had close performance, but MRL was slightly better. ASTRAL and MulRF are next, again with mixed performance (MulRF was better on two datasets and ASTRAL was better on the other two). Finally, ASTRID had the worst performance of all methods - coming in dead last on four of the five datasets. It is worth noting that all but two of the datasets produced distance matrices with missing entries, and ASTRID did better than ASTRAL on one of the two datasets (marsupial) that produced a complete distance matrix.

### 3.4 Topological accuracy

Since the true supertree is not known for the biological datasets, we evaluate topological accuracy only on the simulated datasets red (Table 2). All methods improved in accuracy with the increase in the scaffold density, so that error rates were generally highest for 20%-scaffolds and lowest for 100%-scaffolds. The differences between methods on the 100%-scaffolds were generally small, but there were large differences under the other conditions. ASTRID had very poor accuracy except for those with 100%-scaffolds, and MulRF and PluMiST also had poor accuracy with the lower density scaffolds.

**Table 2:** Supertree topology estimation error on simulated datasets, measured using the Robinson-Foulds error rate, expressed as a percentage

Method	100	100	100	100	500	500	500	500	1000	1000	1000	1000
Scaffold %	20	50	75	100	20	50	75	100	20	50	75	100
# Replicates	9	10	10	10	8	10	10	10	10	10	10	10
ASTRAL	**11.7**	14.0	11.6	10.0	15.3	14.8	12.7	11.2	16.9	15.7	13.6	11.6
ASTRAL-enhanced	11.8	**13:1**	11.5	10.0	14.8	14.1	12.6	11.2	16.3	15.1	13.5	11.6
ASTRID	15.8	18.7	17.1	9.6	26.0	50.1	45.4	10.5	35.6	58.1	52.0	11.2
MRL	13.6	13.6	11.2	10.8	15.4	14.3	12.1	11.2	17.4	15.1	13.5	12.2
MulRF	22.1	26.0	15.3	9.3	46.9	40.3	27.4	12.6	–	–	–	–
PluMiST	25.9	16.6	11.5	9.3	35.4	29.5	22.4	10.9	–	–	–	–
FastRFS-basic	13.5	14.3	10.5	9.1	14.5	14.3	12.4	11.1	17.3	15.6	13.5	12.0
FastRFS-enhanced	13.5	13.4	10.6	9.3	14.3	13.9	12.0	10.8	16.7	15.1	13.4	11.8

The best result for each model condition is boldfaced. No results are shown for PluMiST or MulRF on the 1000-taxon simulated datasets due to running time limitations for these methods. Results are averaged over the completed replicates.

The remaining methods (MRL, the two ASTRAL versions and the two FastRFS versions) were fairly close in accuracy. However, MRL was never more accurate than FastRFS-enhanced, and was only the top performing method for one model condition (where it tied with FastRFS-enhanced). ASTRAL-enhanced was more accurate than ASTRAL on 8 conditions, tied on 1 condition, and less accurate on 3 conditions. FastRFS-enhanced was more accurate than FastRFS-basic on 9 model conditions, tied on 1 condition, and worse on 2 conditions. FastRFS-enhanced was more accurate than ASTRAL-enhanced on 8 of the 12 model conditions, tied on 1 condition and worse on 3 conditions.

FastRFS-enhanced was the top performing method on 5 of the 12 model conditions; the next best performing method was ASTRAL-enhanced, which was the top performing method in 3 of the 12 model conditions. Thus, overall FastRFS-enhanced provided the best accuracy of the tested supertree methods. These results, and especially the pairwise comparisons, suggest that optimizing the Robinson-Foulds Supertree criterion (minimize RF distance) is better than optimizing the ASTRAL criterion (minimize quartet distance) for supertree estimation, and that adding bipartitions from MRL (and from ASTRID if its internode distance matrix is complete) also tends to improve accuracy.

### 3.5 Running time

**Fig. 2:**
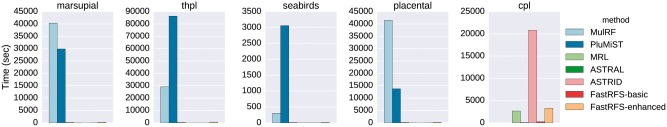
Sequential running times (in seconds) on biological data of supertree methods. MulRF and PluMiST could not be run on the CPL dataset, due to its large size; hence no values are shown for those methods on that dataset

Figure 2 shows running times on the biological datasets. MulRF and PluMiST took the most time, each typically requiring hours where FastRFS-basic, MRL and ASTRAL completed in well under a minute (and sometimes in just a few seconds). MRL and FastRFS-enhanced were the next most computationally intensive, but were sometimes fast, and finally ASTRAL, ASTRID and FastRFS-basic were the fastest, often completing in just seconds. As an example, the running times on the largest dataset on which all the methods completed (THPL, with 558 taxa) showed substantial differences between methods: PluMiST used 86 400 s (i.e. 24 h), MulRF used 29 160 (i.e. 8.1 h), FastRFS-enhanced used 615 s (just over 10 min), MRL used 575 s (i.e. just under 10 min), and ASTRID, ASTRAL and FastRFS-basic used under 20 s.

The size of *X* impacts the running time for FastRFS, and ranged from 1155 to 20 233 for FastRFS-basic and from 2485 to 48 313 for FastRFS-enhanced. The most computationally intensive dataset for FastRFS-enhanced is the CPL dataset, which maximizes both the number of taxa and |X|; however, FastRFS-enhanced completed on this dataset in 3282 s (i.e. under an hour). The majority of the time for FastRFS-enhanced is spent computing the MRL tree; the other parts of the analysis (i.e. computing the ASTRID matrix and potentially the ASTRID tree, computing the constraint set from ASTRAL, and running the DP algorithm) takes very little time (typically less than a minute).

ASTRID’s running time was highly variable, but the running time is high only for large datasets with missing entries in the distance matrix. The reason is that when the matrix has missing entries, ASTRID must use BIONJ* (which takes $\Theta (n^{3})$ time) instead of FastME (which takes $\Theta (n^{2})$ time). For example, ASTRID used about 6 h on the CPL dataset (the only biological dataset with these missing entries), but completed in just seconds on all the other datasets.

## 4 Conclusions

Supertree estimation is a basic bioinformatics challenge that is necessary for the construction of large phylogenies as well as for enabling statistical phylogeny estimation methods to be applied to large datasets. While many methods have been developed to compute supertrees, very few have been able to provide good accuracy on datasets with many hundreds or thousands of species.

The FastRFS methods presented here (i.e. the basic and enhanced versions) are fast and effective techniques to find solutions to the NP-hard Robinson-Foulds Supertree (RFS) problem. FastRFS-enhanced in particular nearly always finds better solutions than PluMiST and MulRF, the leading methods for RFS, and does so in much less time. FastRFS relies upon a dynamic programming algorithm to find an exact solution to its optimization problem within a constrained search space, a strategy introduced in Hallett and Lagergren (2000) and that is quite different from the heuristic search techniques used by most phylogeny estimation methods. Thus, while FastRFS, PluMiST and MulRF all seek to optimize the same criterion, FastRFS is guaranteed to find an optimal solution within its constraint space but cannot return any tree that is not within the constraint space, while PluMist and MulRF are not guaranteed to find an optimal solution within any search subspace but have access to the entire treespace. Thus, our study suggests that exactly solving an optimization problem within a constrained search space may be a better approach than being able to search a larger space, as long as the constrained space is selected carefully. However, our study also shows that expanding the constraint set beyond the input set of source trees can be highly beneficial in terms of finding good solutions to NP-hard optimization problems.

FastRFS-enhanced also tends to find more accurate tree topologies than the other supertree methods we explored. The improvement in topological accuracy suggests that the Robinson-Foulds Supertree problem is a good approach to supertree estimation. The explanation for this is likely to be the close relationship between the Robinson-Foulds Supertree problem and the Maximum Likelihood Supertree problem (Bryant and Steel, 2009), which models source tree discord based on the topological distance to the true supertree (Steel and Rodrigo, 2008). Thus, although a Robinson-Foulds Supertree is not guaranteed to be identical to a Maximum Likelihood Supertree, good solutions to one problem are likely to be good solutions to the other (Bryant and Steel, 2009). Hence, FastRFS may be a good heuristic for the Maximum Likelihood Supertree problem, and this may explain its good accuracy.

There are many directions for future work. For example, since FastRFS by design can only search within the space defined by its constraint set, finding better constraint sets may provide additional improvements. Alternatively, FastRFS-enhanced may provide a good starting tree for PluMiST and MulRF, which are able to search an unconstrained search space. In addition, FastRFS-enhanced may be a good initial tree for Bayesian supertree methods (Akanni *et al.*, 2015a,b; Martins *et al.*, 2016) or heuristic searches for Maximum Likelihood Supertrees (Akanni *et al.*, 2014). Also, like most supertree methods, FastRFS currently only works with inputs where each source tree has at most one copy of each leaf; methods like MulRF are designed to handle inputs of source trees that represent gene trees, and so can have multiple copies of each species (arising from duplication-loss scenarios). We will modify FastRFS to be able to work with such source tree inputs.

## Supplementary Material

Supplementary Data
