# Supplementary material for: FastRFS: fast and accurate Robinson-Foulds Supertrees using constrained exact optimization
Source: Bioinformatics. 2016 Sep 23;33(5):631–9. doi: 10.1093/bioinformatics/btw600 (PMC5870905; doi:10.1093/bioinformatics/btw600)
Supplement: Supplementary Data [file btw600_supp.pdf]

Supporting Data for FastRFS: Fast and Accurate Robinson-Foulds Supertrees using Constrained Exact Optimization

Pranjal Vachaspati and Tandy Warnow

September 3, 2016

1 Size of the constraint set

FastRFS-enhanced uses a larger constraint space than FastRFS-basic. Table 1 shows the sizes of the constraint sets X for the five biological datasets that are added to the search spaces for FastRFS-enhanced and ASTRAL-enhanced.

2 Commands

Commands for the tree estimation software are provided below:

MulRF: We ran MulRF version 1.2 ten times, and the tree with the best optimization score was used. The command was:

```
MulRFSupertree -i <input file name> -o <output file name>
```

PluMiST: We ran PluMiST version 1.1. Since we found PluMiST's stopping condition caused it to run for too long, we allowed PluMiST to run for a limited amount of time (1 hour for 100-taxon simulated dataset; 5 hours for 500-taxon simulated dataset; 12 hours for the the seabird, mammalian, and placental datasets, and 24 hours for the THPL dataset). In all cases, this was at least as long as the other methods took to run and in most cases substantially longer. Reported running times are the times of the last iteration that successfully completed before the cutoff. The command used for PluMiST was

```
python plumist.py -s <input file name> -o <output file name>
```

Method	Seabirds	Placental	Marsupial	THPL	CPL
FastRFS-basic	1155	6907	10251	11109	20233
FastRFS-enhanced	2485	12937	15443	17811	48313

Table 1: Sizes of the set X on biological datasets

MRL: We also ran matrix representation with likelihood (MRL), in which a maximum-likelihood tree is estimated on an MRP matrix. We generated MRP matrices with mrpmatrix, available at `github.com/smirarab/mrpmatrix`:

```
mrpmatrix <input file> <output matrix file> -dna
```

We estimated MRL trees with RAxML version 8.2.4 with command line

```
RAxML -m BINGAMMA -p 12345 -n <run name> -s <matrix file>
```

ASTRID: To run ASTRID, we used the command line

```
ASTRID -i <gene tree file> -o <output file>
```

ASTRAL: To run ASTRAL, we used the command line

```
java -jar astral.4.7.8.jar -i <gene tree file> -o <output file>
```

To run ASTRAL-enhanced, we used

```
java -jar astral.4.7.8.jar -i <gene tree file> -o <output file> -e
<extra trees>
```

where the extra trees file contained the MRL tree or the MRL tree and the ASTRID tree, depending on whether or not the ASTRID distance matrix was complete.

FastRFS-basic: To run FastRFS, we used the command line

```
wASTRAL -c FastRF  -g <gene tree file> -o <output file>
-a /path/to/astral.4.7.8.jar
```

FastRFS-enhanced: To run the enhanced version of FastRFS, we used the command line

```
wASTRAL -c FastRF  -g <gene tree file> -o <output file>
-a /path/to/astral.4.7.8.jar -e <extra trees> --extraextra
```

This runs the clade selection portion of ASTRAL three times to get the constraint set. First, it runs with the input trees as gene trees and the extra trees as extra trees. Second, it runs with the extra trees as the gene trees. Finally, it runs with input and extra trees combined as gene trees. The union of these outputs is used as the clade set for FastRFS.

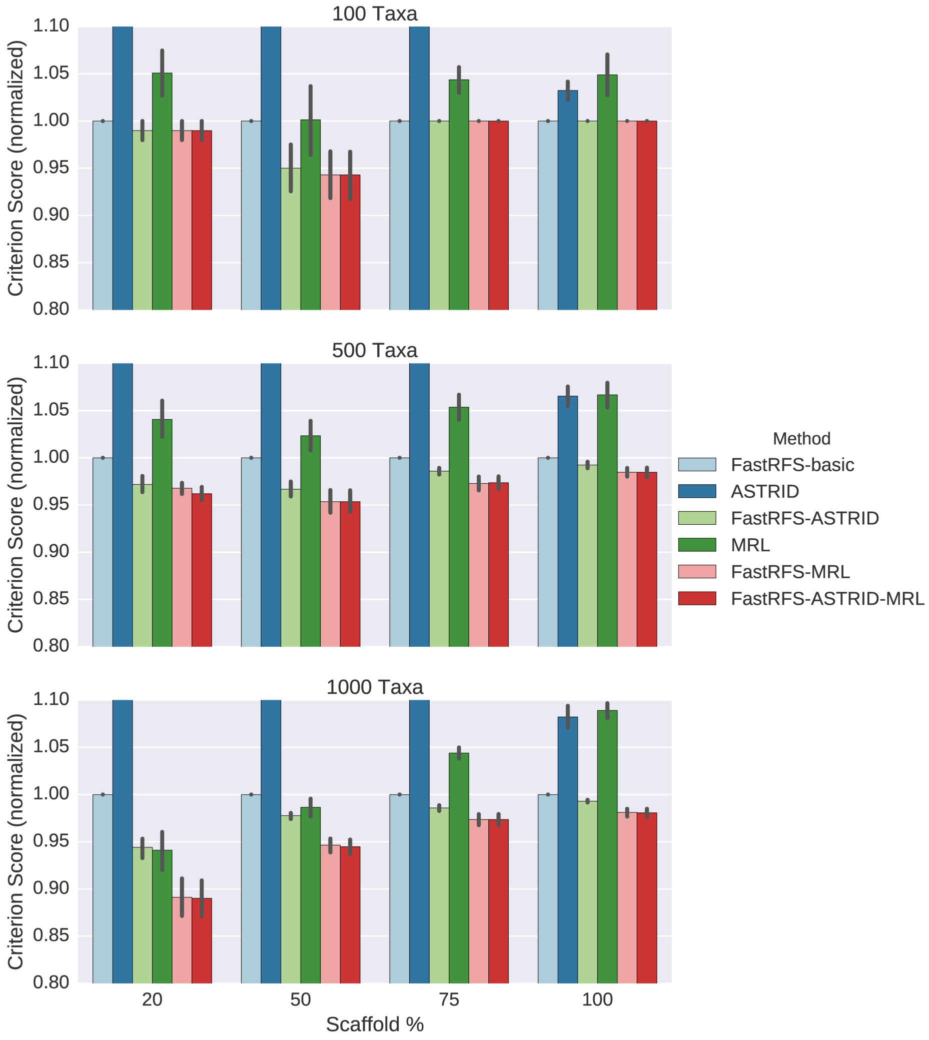

Figure 1: Comparison of FastRFS variants criterion scores on simulated data. Scores are normalized by dividing by the FastRFS-basic score; FastRFS-basic has a score of 1.

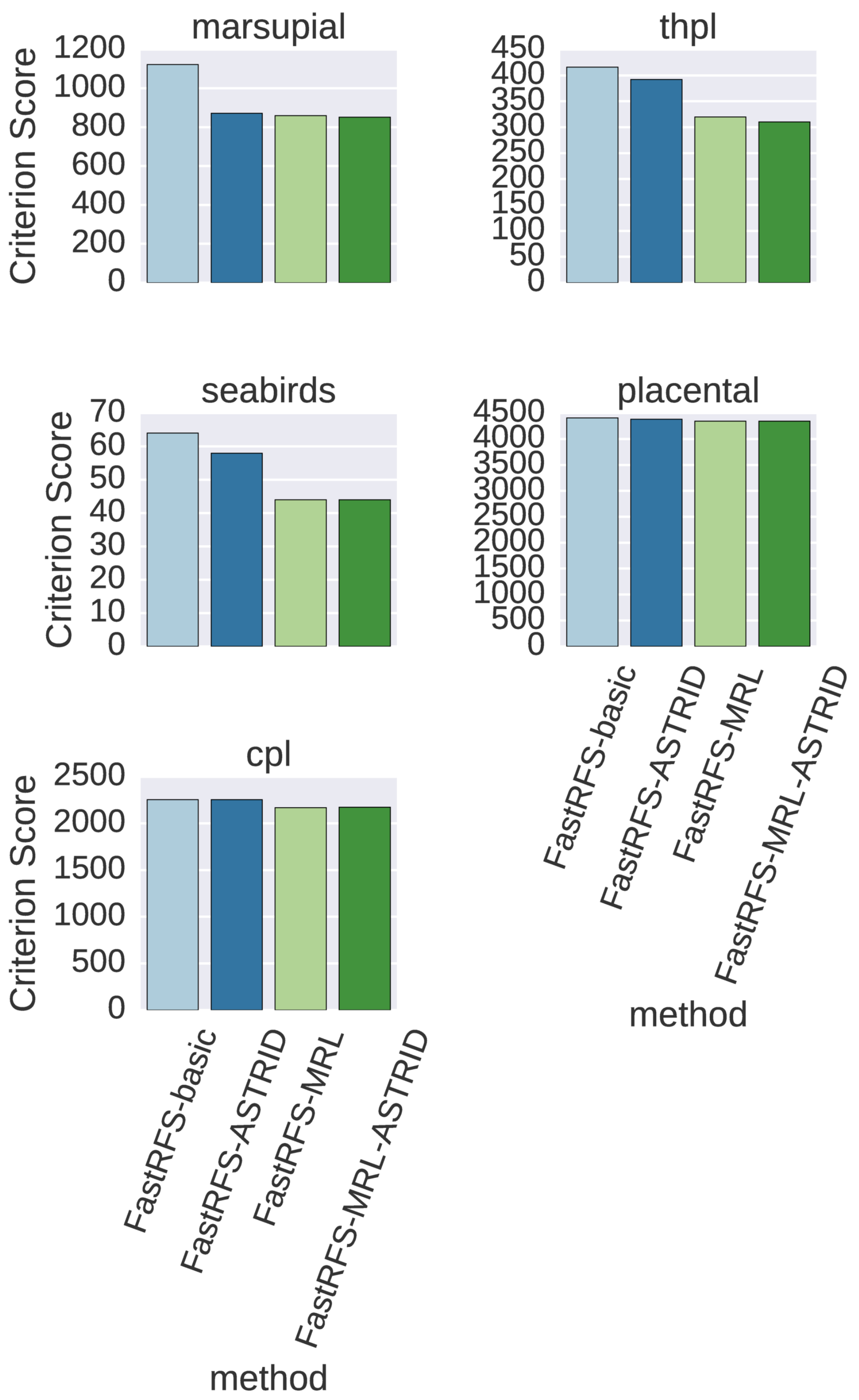

Figure 2: Comparison of FastRFS variants on biological data
